# Abrupt change from moderate positive to colossal negative thermal expansion caused by imidazolate composite formation

**DOI:** 10.1007/s10853-022-07360-z

**Published:** 2022-06-20

**Authors:** Sanja Burazer, Lukáš Horák, Yaroslav Filinchuk, Radovan Černý, Jasminka Popović

**Affiliations:** 1 Department of Condensed Matter Physics, Charles University, Ke Karlovu 5, 121 16, Prague 2, Czech Republic; 2 Institute of Condensed Matter and Nanosciences, Université Catholique de Louvain, Place L. Pasteur 1, 1348 Louvain-la-Neuve, Belgium; 3 Laboratory of Crystallography, DQMP, University of Geneva, Quai Ernest-Ansermet 24, CH-1211 Geneva, Switzerland; 4 Laboratory for Synthesis and Crystallography of Functional Materials, Division for Materials Physics, Ruđer Bošković Institute, Bijenička 54, 10000 Zagreb, Croatia

## Abstract

This work describes temperature-induced crystallization processes and reaction mechanisms occurring in the borohydride-imidazolate system. In the course of thermal evolution, crystal structures of two novel bimetallic imidazolates AMnIm_3_ (A = Na, K) were solved using synchrotron radiation powder diffraction data. Both the alkali metal cation and the Mn cations exhibit distorted octahedral coordination while each imidazolate is surrounded by two alkali metal and two manganese atoms. Extensive study of the thermal expansion behaviour revealed that the expansion of the bimetallic imidazolates does not proceed uniformly over the entire temperature range but rather abruptly changes from a colossal negative to a moderate positive volume expansion. Such behaviour is caused by the coherent intergrowth of the coexisting phases which form a composite, a positive lattice mismatch and a tensile strain during the coexistence of NaMIm_3_ (M = Mg and Mn) and NaIm or HT-NaIm. Such coherent coalescence of two materials opens the possibility for targeted design of zero thermal expansion materials.

**Graphical abstract:**

Crystal structures of AMnIm_3_ (A = Na, K) were determined. Coherently intergrown NaMIm_3_/NaIm (M = Mg, Mn) present colossal negative thermal expansion.

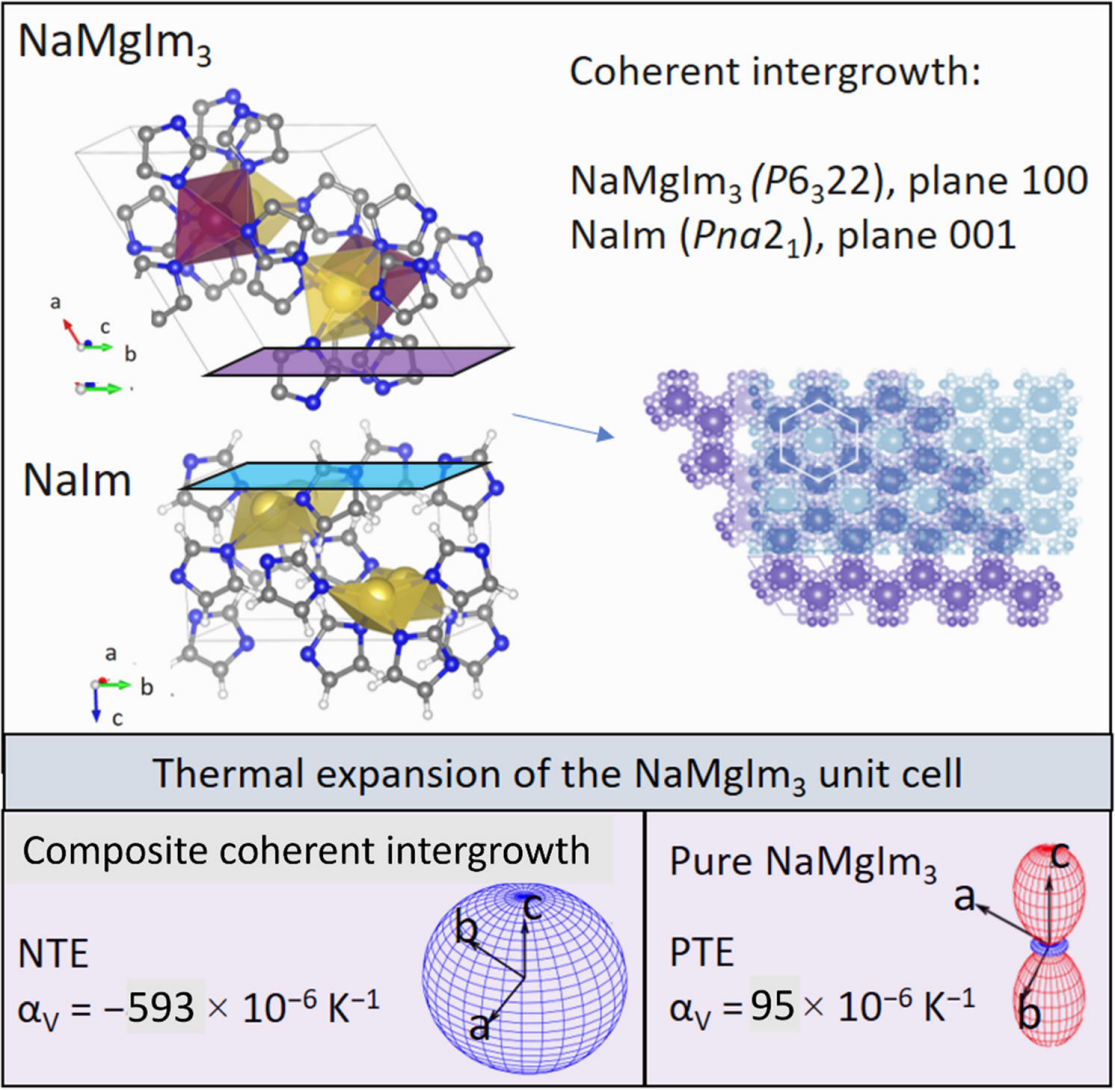

**Supplementary Information:**

The online version contains supplementary material available at 10.1007/s10853-022-07360-z.

## Introduction

Typically, many materials exhibit positive thermal expansion (PTE). Nowadays, the materials science community is intrigued by the uncommon phenomenon that occurs in some types of materials, causing them to contract when heated, i.e. to exhibit a negative thermal expansion (NTE). Such thermal behaviour enables fabrication of composites materials with a tailored coefficient of thermal expansion, namely, zero expansion [1]. Zero expansion could prevent performance deterioration or even failure of devices caused by large differences in expansion coefficients [2]. In some typical NTE materials, such as ZrW_2_O_8_ [3], Cd(CN)_2_ [4] and ScF_3_ [5] with linear coefficients ranging from *α* =  − 14 × 10^−6^ K^−1^ for ScF_3_ [6] to *α* =  − 34 × 10^−6^ K^−1^ for Cd(CN)_2_, contraction upon temperature increase has been reported [7]. For molecular materials, the NTE behaviour usually depends on many factors, including transverse vibrational modes, geometric flexibility, host‐guest interaction, spin states, molecular packing arrangements, and molecular configurations [8]. Various metal–organic frameworks (MOFs) show NTE values of − 27 × 10^−6^ K^−1^ ≤ *α* ≤  − 11 × 10^−6^ K^−1^ [9]. To emphasize the differences from typical framework behaviour, the term “colossal” is used for coefficients ≥ 100 × 10^−6^ K^−1^ [10–12]. As an example, the perovskite PbVO_3_ exhibits *α* =  − 590 × 10^−6^ K^−1^ albeit in a short temperature interval of only 30 K [13]. Polycrystalline materials, especially those prepared by high energy ball milling, contain numerous microstructural imperfections. Numerous properties, such as microstructural, optical, electrical and magnetic are typically strongly temperature dependent but in some cases, the thermal expansion behaviour can also be extremely temperature dependent in the sense that a material exhibiting PTE in one temperature range can abruptly change its thermal expansion behaviour and end up with NTE in another temperature range [14–19]. One such example is the cyanide-bridged Fe^III^–Co^III^ complex; a compound that exhibits an immense volumetric coefficient of thermal expansion of 1498 × 10^−6^ K^−1^ (between 180 and 240 K) followed by a negative volumetric expansion coefficient of − 489 × 10^−6^ K^−1^ in the temperature range of 300–350 K [20]. The formation of the two-phase composite with PTE and NTE materials is an effective way to obtain (near) zero-thermal expansion materials, especially needed and utilized in the fields of mechanics and aerospace [21–33]. While intrinsic properties, such as electronic, ferroelectric or magnetic behaviour sensitive to microstructure, are considered as cause of thermal expansion, the influence of surface and interface on thermal expansion still needs more research [1, 34–36].

In an endless sea of metal organic frameworks (MOFs), experiencing explosive growth due to their ample chemical versatility, exceptional porosity and a wide range of potential applications including gas storage, separation, and catalysis, zeolitic imidazolate frameworks (ZIFs) are extremely popular [37]. They contain a combination of tetrahedral nodes which incorporate electronic properties of the transition metal ions and bent linkers that mimic the structural features of zeolites [38]. While various metal centres have been vastly studied, alkali metals have been left out of focus possibly due to the fact that NaIm, KIm and LiIm do not form porous frameworks, yet their dense and hypercoordinated structures have been observed [39, 40]. Nevertheless, our recent work has shown that the high-temperature polymorph of NaIm is capable of forming a porous structure [41]. On the other hand, magnesium imidazolate has been considered as a promising complexing agent due to the nature of the magnesium ion and its coordination chemistry. The studies carried out by Safin and collaborators revealed that the freshly synthesized MgIm_2_ is amorphous but annealing at higher temperatures leads to crystalline and porous MgIm_2_ [42]. At this point, it seemed opportune to extend the study by combining both alkali and alkaline earth metal centres, thus we recently reported the first bimetallic imidazolates containing alkali and alkaline earth metals, NaMgIm_3_ and KMgIm_3_ [40]. Both compounds are isostructural and crystallize in the hexagonal *P*6_3_22 space group. The crystal packing reveals channels with the empty volume calculated from the contact surface which is about 30 Å^3^. Although the cage size recorder, ZIF-412 [43] with 38.1 Å, is able to selectively bind large volume volatile organic compounds, octane and p-xylene, AMgIm_3_ with the cage diameter of 6.6 Å can still be applicable in gas sorption/separation processes. On the other hand, the preparation of manganese imidazolates remains a real challenge, probably due to the difficulties in forming an undistorted tetrahedral Mn^2+^ − 4 N geometry which is preferred in ZIFs. According to the Cambridge Crystallographic Data Centre database, only a few compounds with an undistorted tetrahedral Mn^2+^ − 4 N geometry are known [44–47]. It has been discussed in the literature that [Mn(Im)_2_·2(ImH)] (ImH = imidazole) with distorted tetrahedral Mn^2+^ − 4 N (N − Mn − N is 99.86 − 126.45°) was synthesized from Mn_2_(CO)_10_ and melting ImH but this reaction produces CO and H_2_ gases as by-products along with formation of the kinetically favoured framework [48]. Therefore, the formation of crystalline manganese imidazolate frameworks still remains an unsatisfied task and requires detailed research.

In this work, we report two new crystal structures of bimetallic imidazolates, AMnIm_3_ (A = Na, K) which were solved using powder X-ray diffraction data measured at a synchrotron facility. The original intent of this work was to explore the possibility of forming coordination frameworks containing both the imidazolate and complex hydride anions as ligands. Although such a metal-hydride organic framework was not obtained, temperature-assisted structural and microstructural analysis allowed a deeper understanding of the crystallization processes and reaction mechanisms occurring in the borohydride-imidazolate system. Extensive study of the thermal expansion behaviour revealed that the expansion of the bimetallic imidazolates does not proceed uniformly over the entire temperature range but rather abruptly changes from a colossal negative to a moderate positive volume expansion. Such behaviour is caused by the coherent intergrowth of the coexisting phases which form a composite, a positive lattice mismatch and a tensile strain during the coexistence of NaMIm_3_ (M = Mg and Mn) and NaIm or HT-NaIm.

## Experimental section

### Synthesis

Four neat grinding mechanochemical reactions of Mg(BH_4_)_2_ or Mn(BH_4_)_2_ and AIm (A = Na, K) were conducted using a Planetary Micro Mill Fritsch Pulverisette 7 premium line. Reactants, together with stainless steel balls (*d* = 5 mm), were loaded into a stainless steel vial (25 ml) under inert conditions. Balls-to-sample mass ratio amounted to 25:1 in each reaction. Ball milling was performed at 550 rpm (10 min of milling, followed by a 5 min rest time; repeated 12 times). Molar ratios of reactants are given in Table 1. Anhydrous magnesium borohydride, Mg(BH_4_)_2_ (99.99%) as well as anhydrous manganese borohydride, Mn(BH_4_)_2_ were purchased from Sigma-Aldrich, while imidazolates AIm (A = Na, K) were prepared by the procedure reported elsewhere [39]. All handling and manipulation of the chemicals were performed in an argon-filled glovebox. All solvents have been dried on a vacuum line prior to the mechanochemical reactions.

**Table 1:** Reactants used for the mechanochemical synthesis

	Molar ratio of reactants
S1	Mg(BH_4_)_2_ and NaIm = 1:6
S2	Mg(BH_4_)_2_ and NaIm = 1:2
S3	Mn(BH_4_)_2_ and NaIm = 1:6
S4	Mn(BH_4_)_2_ and KIm = 1:6

### X-ray powder diffraction (XRPD) at RT

XRPD measurements at room temperature (RT) were performed using a Stoe IPDS-P diffractometer with monochromated CuKα1 radiation (λ = 1.5406 Å) and a curved image plate detector, in Debye–Scherrer geometry. Air-sensitive samples were mounted in a glovebox in 0.8 borosilicate capillaries sealed with vacuum grease. Data were collected at RT, in 2*θ* range: 2 − 100° with counting time of 40 s/step. XRPD patterns are shown in Fig. S1 in the supporting information.

### Synchrotron radiation X-ray powder diffraction (SR-XRPD) at HT

Synchrotron radiation experiments were done at the beamline BM01, SNBL at the ESRF, Grenoble, France. High temperature *in-situ* powder diffraction (HT-XRPD) data were collected with measurement parameters: λ_S1,S2_ = 0.8187 Å, λ_S3,S4_ = 0.7225 Å, sample rotation 0 − 40° and X-ray exposure time of 40 s. The air sensitive samples were mounted in 0.5 mm borosilicate capillaries and closed with vacuum grease. The Dectris Pilatus 2 M detector was used for recording 2D powder data at the sample to detector distances of 400 (samples S1 and S2) or 300 (samples S3 and S4) mm. The local program Bubble was used for integration of the 2D images [49]. Samples were heated by a heat blower from RT to 350 °C (with a 5 °C/min heating rate).

### Structure solution and microstructural analysis

SR-PXD data were used for structure determination of new compounds. Indexing, space group determination and structure solution was carried out using Fox program [50]. Fullprof program [51] was used for Rietveld refinement [52] of the structural model. The visualizations of the crystal structures were made by programs VESTA [53] and Mercury [54]. Diffraction profiles of thermally treated samples were analysed by Rietveld refinement implemented in whole-powder-pattern modelling (WPPM) program MSTRUCT that is capable to determine the microstructure parameters such as micro-strain or crystallite size [55]. Software PASCal is used for calculation and visualization of thermal expansion behaviour [56].

## Results and discussion

### Thermal evolution of crystallization processes in borohydride-imidazolate system

**Figure 1:**
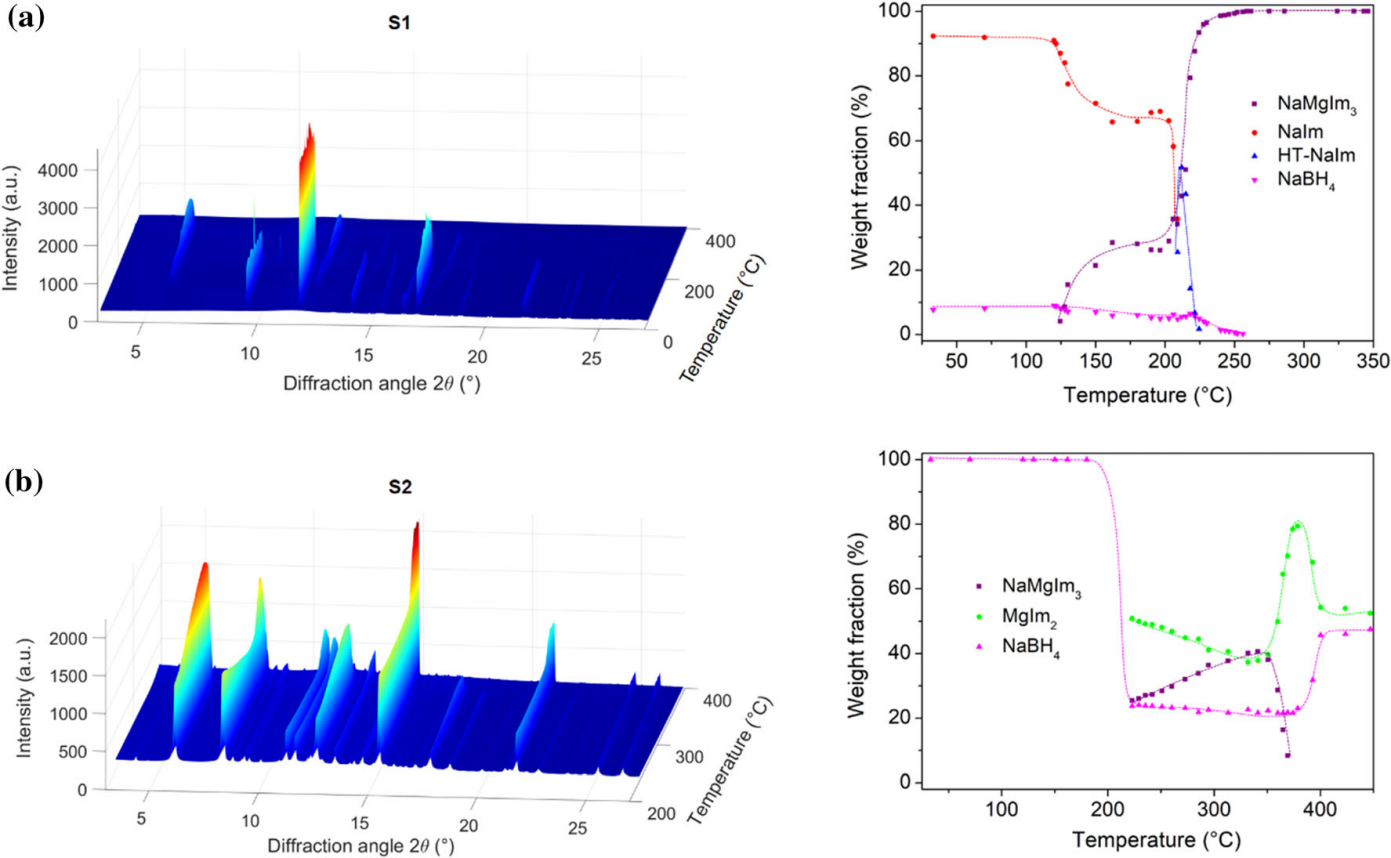
*In-situ* variable temperature XRPD data and changes in composition as a function of temperature for sample: **a** S1 and **b** S2. The orientation of the plot is chosen in such a way that no important data are hidden. Dashed lines represent guidelines for eye only

The bimetalic imidazolate, NaMgIm_3_, reported in our previous article [40], was prepared starting from Mg(BH_4_)_2_:NaIm in a 1:6 ratio. To gain a deeper understanding of the crystallization processes in the borohydride-imidazolate system an additional mechanochemical experiment was carried out using a different ratio of reactants, namely, Mg(BH_4_)_2_:NaIm in the 1:2 ratio. The 1:2 ratio (with less imidazolate than required for the formation of NaMgIm_3_) was chosen to explore the possibility of forming bimetallic imidazolates that have a different stoichiometry than that reported. Interestingly, as the discussion will show, mechanochemical reaction performed in 1:2 ratio also resulted in the formation of NaMgIm_3._ Nevertheless, as shown in Fig. 1, the temperature-induced structural evolution of the phases in samples S1 and S2 which occurs before and during the crystallization of NaMgIm_3,_ depends significantly on the initial amounts of imidazolate and borohydride.

At room temperature (RT), both mechanochemical reactions, performed in a 1:6 (S1) and 1:2 (S2) ratio, induce an ion exchange reaction leading to formation of NaBH_4_. In both cases, the magnesium imidazolate, as the second product of the exchange reaction, becomes amorphous. In the case of the 1:6 reaction, where NaIm was used in excess, a certain amount of NaIm remained unreacted. Upon thermal treatment, the main difference is observed in terms of crystallization temperature of the bimetallic imidazolate; for an initial ratio of 1:2, the crystallization of NaMgIm_3_ shifts to a higher temperature (from ~ 120 °C, in the case of 1:6, to ~ 220 °C for the 1:2 ratio). Such a pronounced difference in crystallization temperature is, in fact, a consequence of the different pathways, content-wise, that precede the crystallization of NaMgIm_3_.

The mechanochemical reaction at RT can be described by Eq. 1:

1 $$ {\text{Mg}}\left( {{\text{BH}}_{{4}} } \right)_{{2}} + {\text{2NaIm}} \to {\text{2NaBH}}_{{4}} + {\text{MgIm}}_{{{2}({\text{amorphous}})}} $$

The subsequent thermal treatment proceeds by different reaction routes depending on the NaIm excess: at high NaIm excess (sample S1), the bimetallic imidazolate NaMgIm_3_ is formed at *T* = 125 °C by the reaction of the metal imidazolates (Eq. 2):

2 $$ {\text{NaIm}} + {\text{MgIm}}_{{{2}({\text{amorphous}})}} \to {\text{NaMgIm}}_{{3}} $$

Upon further increase in temperature, NaIm undergoes the phase transition (at *T* = 209 °C) to its high-temperature polymorph. HT-NaIm melts above 224 °C (which is consistent with [41]), while NaBH_4_ melts above 255 °C. Above 255 °C up to 346 °C the only crystalline phase in the sample is NaMgIm_3_. This sample was heated up to 400 °C where NaMgIm_3_ melts or decomposes (see sample S2 below) and cooled to RT, but NaMgIm_3_ does not recrystallize.

On the other hand, in the sample S2 with starting ratio of 1:2, the mechanochemical reaction follows Eq. 1, but unlike sample S1, the temperature-induced crystallization of NaMgIm_3_, follows the reaction between sodium borohydride and magnesium imidazolate as shown by Eq. 3:

3 $$ {\text{2NaBH}}_{{4}} + {\text{3MgIm}}_{{{2}({\text{amorph}}.)}} \to {\text{2NaMgIm}}_{{3}} + {\text{Mg}}\left( {{\text{BH}}_{{4}} } \right)_{{{2}({\text{amorph}}.)}} $$

MgIm_2_ crystallizes at *T* = 220 °C. Above 370 °C, the crystalline NaMgIm_3_ melts or decomposes. Sample S2 was heated up to 450 °C and at this temperature MgIm_2_ and NaBH_4_ are present as crystalline phases in the system. It is interesting to note that the melting point of pure NaBH_4_ is about 400 °C, so the higher melting point may be caused by the formation of an amorphous peritectic phase NaMg(BH_4_)Im_2_.

**Figure 2:**
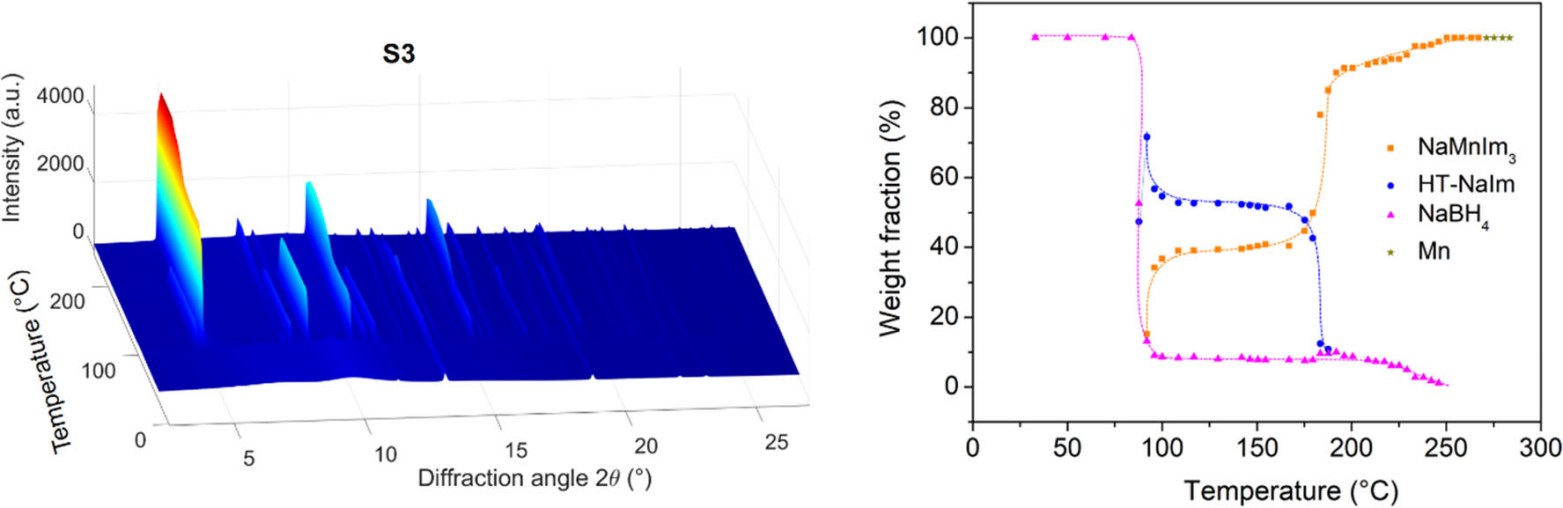
*In-situ* variable temperature XRPD data and changes in composition as a function of temperature for sample S3. The orientation of the plot is chosen in such a way that no important data are hidden. Dashed lines represent guidelines for eye only

Our research was further extended to reactions between 3*d* transition metal borohydride Mn(BH_4_)_2_ and alkali metal (A = Na, K) imidazolates. The temperature-induced structural evolution of sample S3 (A = Na) is shown in Fig. 2.

Comparing the temperature-induced structural evolution of magnesium (S1) and manganese (S3) systems, both with the same initial ratio of borohydride to imidazolate of 1:6, reveals similar behaviour, although some important additional observations can be made. In the case of manganese, a mechanochemical reaction of Mn(BH_4_)_2_ and NaIm again resulted in a cation exchange leading to the formation of crystalline NaBH_4_ and amorphous MnIm_2_ (Eq. 4) but unlike in the case of magnesium, the excess of NaIm becomes amorphous.

4 $$ {\text{Mn}}\left( {{\text{BH}}_{{4}} } \right)_{{2}} + {\text{2NaIm}} \to {\text{2NaBH}}_{{4}} + {\text{MnIm}}_{{{2}({\text{amorphous}})}} $$

Although the milling conditions were identical to those of S1, it is plausible that the greater hardness of Mn(BH)_4_, compared to Mg(BH)_4,_ causes the amorphization of NaIm. As the discussion will show, the observed mechanochemical amorphization of excess NaIm has important implications for the thermodynamic aspects of the phase transition to the high-temperature polymorph. Further thermal treatment (at *T* ~ 90 °C) led to the crystallization of a new phase, later recognized as NaMnIm_3_, according to the reaction (Eq. 5):

5 $$ {\text{2NaBH}}_{{4}} + {\text{3MnIm}}_{{{2}({\text{amorphous}})}} \to {\text{2NaMnIm}}_{{3}} + {\text{Mn}}\left( {{\text{BH}}_{{4}} } \right)_{{2}} $$

Unlike in the case of the magnesium system (Eq. 2), here we observe the presence of the excess NaIm in its crystalline high-temperature form at the temperature of formation of the bimetallic imidazolate. It is particularly interesting to note that crystalline room-temperature NaIm, as present in the case of magnesium system, transforms to HT- crystalline polymorph at *T* = 209 °C, whereas amorphous NaIm, as present in the manganese system, transforms to HT-polymorph at as low as 87 °C. With further increase in temperature, HT-NaIm remains crystalline up to 188 °C, while NaBH_4_ remains crystalline up to 246 °C. At higher temperatures, only NaMnIm_3_ is present in the sample and remains stable up to 280 °C. Above 272 °C, NaMnIm_3_ decomposes and a small amount of metallic Mn crystallizes.

**Figure 3:**
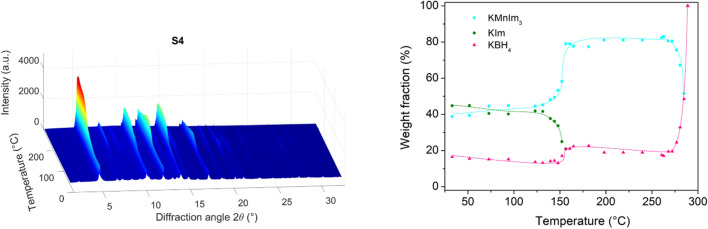
*In-situ* variable temperature XRPD data and changes in composition as a function of temperature for sample S4. The orientation of the plot is chosen in such a way that no important data are hidden. Dashed lines represent guidelines for eye only

Finally, the mechanochemical reaction of Mn(BH_4_)_2_ and KIm (in a 1:6 ratio) was investigated (sample S4, Fig. 3).

While one would have expected similar products to the reaction between Mn(BH_4_)_2_ and NaIm, structural analysis showed that the new bimetallic KMnIm_3_ had already crystallized, during ball-milling, according to the reaction which is equivalent to the sum of reactions (1) and (2):

6 $$ {\text{Mn}}\left( {{\text{BH}}_{{4}} } \right)_{{2}} + {\text{3KIm}} \to {\text{2KBH}}_{{4}} + {\text{KMnIm}}_{{3}} $$

With further temperature increase, the excess KIm decomposes or melts at 155 °C, while KMnIm_3_ remains stable from RT up to 285 °C. Above this temperature, it melts or decomposes and only KBH_4_ remains crystalline in the sample. Equation 1–6 are overall equations for the mechanochemical reactions, while the partial equations encompassing all components present in the samples (including those that do not react with other phases) can be found in the supporting information (Eqs. S1-S7).

The thermal stability of the bimetallic magnesium and manganese imidazolates prepared in a 1:6 ratio is summarized in Table 2. It is observed that potassium manganese imidazolate is swiftly formed during milling at RT, while the additional thermal treatment is required to induce crystallization of the sodium bimetallic imidazolates. Manganese bimetallic imidazolates melt in the temperature range ~ 280–290 °C, while the magnesium compounds remain stable up to ~ 340–350 °C. This finding was expected considering that the degree of ionic *versus* covalent character is greater for the Mg-N bond than in the case of manganese. Since no recrystallization was observed in our experiments upon cooling of any bimetallic imidazolates, we conclude that the compounds decompose before melting.

**Table 2:** Thermal stability of AMIm_3_ (A = Na, K; M = Mg, Mn)

Compound	Stability range (°C)	Recrystallization
NaMgIm_3_	Crystallization: 122 °C Decomposition: 340 °C	No data
NaMnIm_3_	Crystallization: 87 °C Decomposition: 280 °C	No, only Mn is present during cooling back to RT
KMnIm_3_	Crystallization: RT (during milling) Decomposition: 289 °C	No, only KBH_4_ is present during cooling back to RT

### Crystal structures of AMnIm_3_ (A = Na, K)

**Figure 4:**
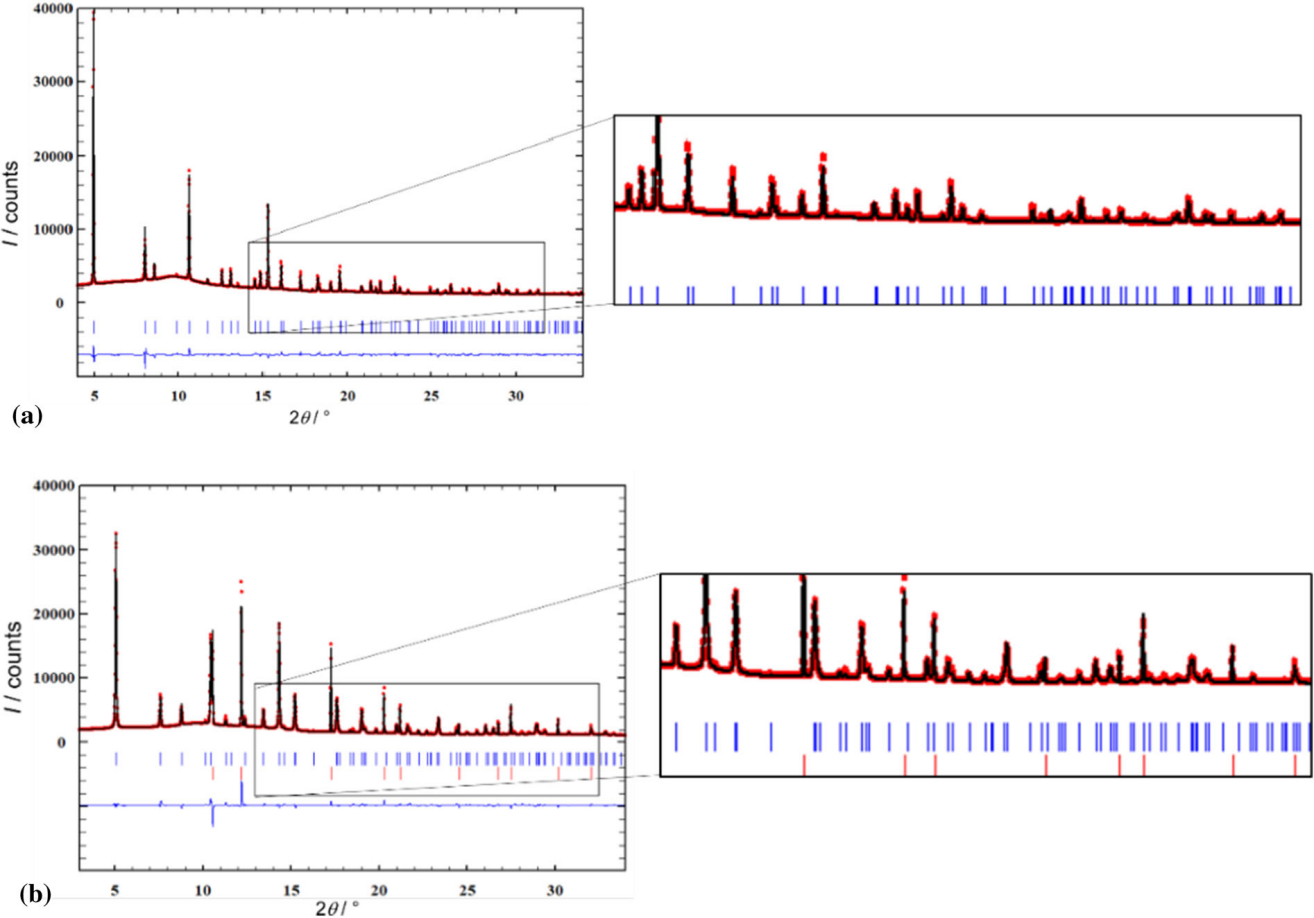
Rietveld refinement of **a** sample S3 used for the structural determination of NaMnIm_3._ Vertical marks represent Bragg reflections of NaMnIm_3_, and **b** sample S4 used for the structural determination of KMnIm_3_. Blue vertical marks represent Bragg reflections of KMnIm_3_ and red represents KBH_4_. Experimental pattern is given as red dots, black curve shows calculated profile and the difference curve is given in blue. Enlarged part of diffraction pattern is shown to illustrate the quality of refinement

Two new crystal structures, AMnIm_3_ (A = Na, K) were solved from synchrotron X-ray powder diffraction data at elevated temperatures. A single phase pattern collected at 200 °C was used for structure determination of NaMnIm_3_, while the crystal structure of KMnIm_3_ was determined from the multiphase pattern collected at 173 °C. The Rietveld refinement plots for NaMnIm_3_ and KMnIm_3_ are given in Fig. 4. Crystal data and summary of structure refinement data are listed in Table 3.

**Table 3:** Crystal data and summary of structure refinement for NaMnIm_3_ and KMnIm_3_

	NaMnIm_3_	KMnIm_3_
Profile function	Pseudo voigt	Pseudo voigt
*R* (profile)/%	4.9	6.0
*R* (weighted profile)/ %	7.7	10.1
*χ*^2^	1.16⋅10^3^	5.16⋅10^3^
Space group	*P*6_3_22	*P*6_3_22
*a*/Å	9.64595(13)	9.42292(15)
*c*/Å	6.57337(9)	7.33233(18)

**Figure 5:**
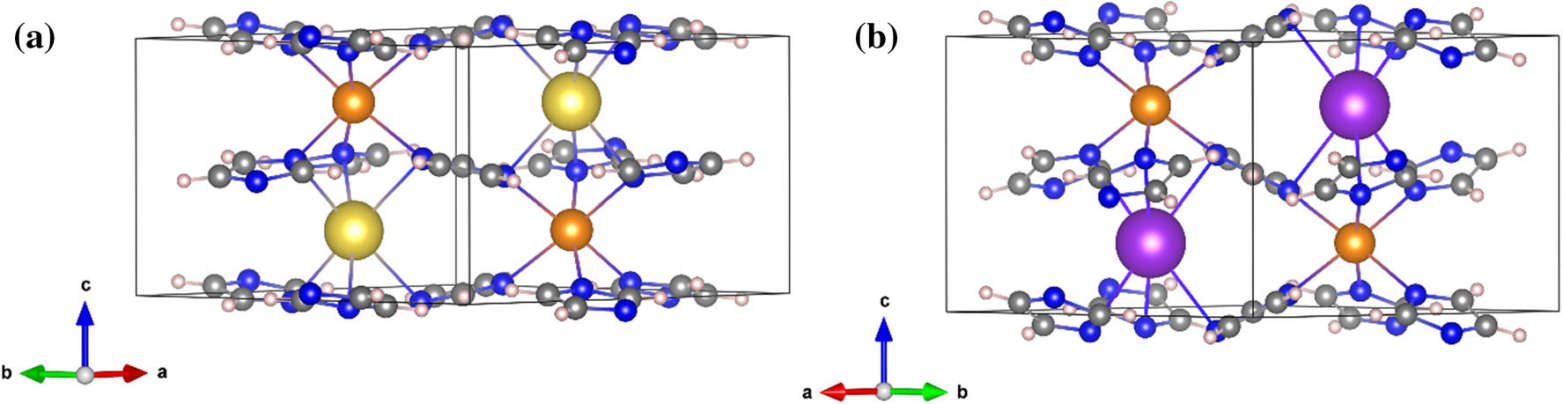
Crystal structure of **a** NaMnIm_3_ and **b** KMnIm_3_. Manganese atoms are shown as orange balls, sodium as yellow, potassium is given in purple, carbon is grey while nitrogen is shown in blue colour

Both compounds crystallize in a hexagonal system (space group *P*6_3_22). The positions of a Na (or K) atom, a Mn atom, and an imidazolate group were varied in Fox using the appropriate antibump restraints in order to determine the structure. The imidazolate ring was treated as a rigid body, only its position and orientation in the unit cell were warried. Interestingly, the structure determination of alkali manganese imidazolates is quite different from the structure solution process which gave a correct structure in the case of alkali magnesium imidazolates. For AMgIm_3_ (A = Na, K), the parallel tempering procedure, with one billion trials per run, was a good choice, but this strategy, when applied to AMnIm_3_ (A = Na, K) resulted in the false minimum structures, with doubled imidazolate rings. Therefore a different global optimization strategy was used for the structure solution of AMnIm_3_ (A = Na, K). The correct structures were obtained by utilizing a small number of runs and trials with each run followed by a least squares refinement procedure [50]. One possible explanation is that the X-ray diffraction contrast between Na^+^ and Mg^2+^ is practically zero, which makes the parameter space in the global optimization simpler, even if the false minima arise [57]. The false minima were eliminated and both the NaMnIm_3_ and KMnIm_3_ structures were validated by BVS calculations [58]. The crystal structures of NaMnIm_3_ and KMnIm_3_ are shown in Fig. 5.

**Figure 6:**
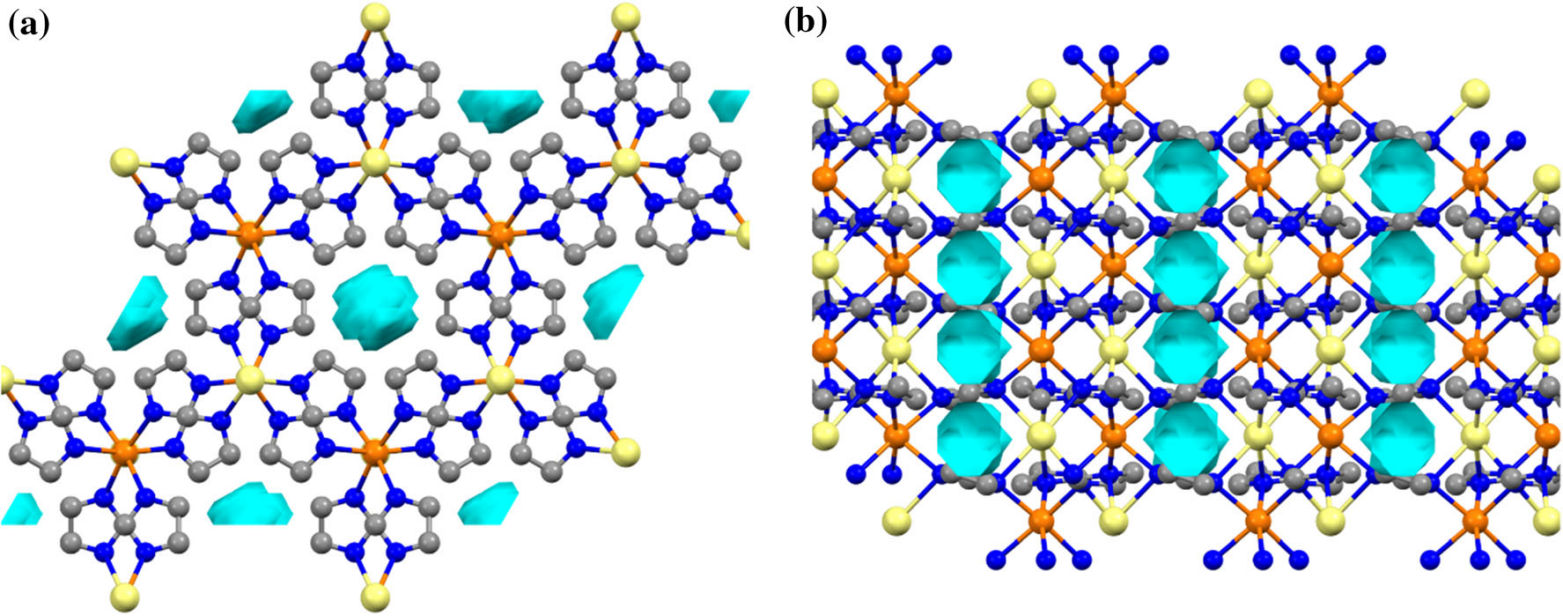
Crystal packing of NaMnIm_3_ in *ab* and *bc* plane. Manganese atoms are shown as orange balls, sodium as yellow, potassium is given in purple, carbon is grey while nitrogen is shown in dark blue colour. Free volume in pores is represented as light blue spheres. Hydrogens are omitted for clarity

In contrast to monometallic NaIm, in which the sodium exhibits tetrahedral coordination [39], in the structure of bimetallic AMnIm_3_ (A = Na, K) both the alkali metal cation and the Mn cations exhibit distorted octahedral coordination. Each imidazolate is surrounded by four metal centres; two alkali metal and two manganese atoms. Although the crystal structures of NaMnIm_3_ and KMnIm_3_ are quite similar a difference is observed with respect to the polyhedra around the alkali atoms; in the case of NaMnIm_3_ the octahedra around the sodium are more distorted than those around the potassium atoms in the case of KMnIm_3_. Along the *c*-direction, the chains consisting of alternating A (A = Na, K) and Mn face-shared octahedra are formed from three imidazole rings via bridging nitrogen atoms. The chains running parallel to *c*-axis are additionally mutually connected by bridging imidazolate anions, forming *zig-zag* networks in the *ab* and *ac* planes, that eventually leads to a three-dimensional network (Fig. 6). It also reveals the presence of channels running along the *c*-direction, and located on the 6_3_ screw axis.

The empty volume of pores calculated by Mercury (Cambridge Crystallographic Data Centre) [54], located in the channels running parallel to *c*-axis, in AMIm_3_ (A = Na, K, M = Mg, Mn) are given in Table 4.

**Table 4:** Bond length distances A − N_im_ and M − N_im_ and volume of pores in AMIm_3_ (A = Na, K, M = Mg, Mn)

Compound	Bond length distances M-N_im_	Empty volume in the pores	
NaMgIm_3_	*d*(Na_oct_ − N_Im_) = 2.5851(2) Å *d*(Mg_oct_ − N_Im_) = 2.3701(2) Å	30.51 Å^3^ (5.9% of unit cell volume) *r* = 1.3 Å	This work
KMgIm_3_	*d*(K_oct_ − N_Im_) = 3.081(3)Å *d*(Mg_oct_ − N_Im_) = 2.329(3) Å	22.62 Å^3^ (4.1% of unit cell volume) *r* = 1.1 Å	Ref [40]
NaMnIm_3_	d(Na_oct_ − N_Im_) = 2.6685(1) Å *d*(Mn − N_Im_) = 2.3722(1) Å	35.19 Å^3^ (6.6% of unit cell volume) *r* = 1.4 Å	This work
KMnIm_3_	*d*(K_oct_ − N_Im_) = 3.031(7)Å *d*(Mn_oct_ − N_Im_) = 2.379(7) Å	30.82 Å ^3^ (5.5% of unit cell volume) *r* = 1.2 Å	This work

Contact surface amounts to 35.2 Å^3^ for NaMnIm_3_ (assuming a spherical probe of *r* = 1.4 Å) and 30.8 Å^3^ for KMnIm_3_ (assuming a spherical probe of *r* = 1.2 Å). For comparison, Mn(BH_4_)_2_ has isolated voids with an estimated volume of 21 Å^3^ [59–61]. It can be seen from Table 4 that NaMnIm_3_ has the largest pore volume among the group AMIm_3_ (A = Na, K; M = Mg, Mn), that amount to 6.6% of the unit cell volume, whereas KMgIm_3_ contains pores covering only 4.1% of unit cell volume. This finding might be related to the more isotropic nature of interactions involving 3* s* orbitals of magnesium compared to directional interactions of 3*d* orbitals in the case of manganese, as well as to the fact that the imidazolate ring is almost parallel to the *ab* plane in the case of the sodium compound, while the potassium compound shows a pronounced ring tilting, leaving less free space. AMnIm_3_ compounds are isostructural with their magnesium analogues reported in our previous paper, AMgIm_3_ (A = Na, K) [40]. That was, in fact, quite expected if one considers the structural similarities between Mn- and Mg-borohydride compounds. The bond between the manganese atom and the nitrogen atom from the imidazolate ring in NaMnIm_3_ [*d*(Mn − N_Im_) = 2.3722(1) Å] is slightly longer than the corresponding bond in NaMgIm_3_ [*d*(Mg − N_Im_) = 2.3701(1) Å]. As it is also the case with borohydrides, larger Mn^2+^ in γ-Mn(BH_4_)_2_ forms a larger unit cell compared to γ-Mg(BH_4_)_2_ (*V*_NaMnIm3_ = 529.674(18) Å^3^, *V*_NaMgIm3_ = 514.55(5) Å^3^). The bond distance *d*(Mn − N_Im_) which is 2.3722(1) Å also indicates the high spin state of manganese in NaMnIm_3_ [62]_._

### Thermal expansion behaviour

Rietveld refinement of the *in-situ* high temperature XRPD data revealed a complex nature of the crystallization pathways of the bimetallic imidazolates but also interesting trends in unit-cell expansion as a function of temperature. Table 5 gives the thermal expansion coefficients α along the *a*, *b* and *c*- directions for bimetallic imidazolates AMIm_3_ (A = Na, K; M = Mg, Mn), as well as for monometallic imidazolates and borohydrides that are also present in different temperature intervals in samples S1-S4. The expansion coefficients were calculated in the temperature range given in Table 5. The temperature ranges do not necessarily represent the entire range in which a particular phase is present. Intervals near crystallization/transformation/decomposition/melting temperature were omitted due to uncertainty in determining unit-cell parameters when the amount of phase is small.

**Table 5:** Thermal expansion coefficients α along the *a*, *b* and *c*- direction for bimetallic imidazolates AMIm_3_ (A = Na, K; M = Mg, Mn), metal imidazolates AIm_x_ (A = Na, K, Mg) and metal borohydrides ABH_4_ (A = Na, K) in samples S1-S4

	α_i_ ($\times {10}^{-6}{\mathrm{K}}^{-1}$)							
	S1			S2	S3		S4	
	NaMgIm_3_			NaMgIm_3_	NaMnIm_3_		KMnIm_3_	
	150–209 °C	212–224.5 °C	227–343.2 °C	222.8–359 °C	92–187.7 °C	191–279.8 °C	33–94 °C	156.4–284.9 °C
*a*	− 181(8)	− 82(7)	− 9(2)	− 19(1)	− 113(1)	− 14(2)	− 16(1)	− 15(1)
*c*	− 210(8)	− 57(5)	34(5)	41(1)	− 56(0)	52(3)	83(7)	117(3)
	NaIm 33–209 °C	HT-NaIm 209–215 °C		MgIm_2_ 222.8–423 °C	HT-NaIm 108–187.7 °C		KIm 33–152.2 °C	
*a*	80(3)	113(19)		33(1)	7(1)		84(2)	
*b*	− 27(1)							
*c*	104(5)	197(0)		− 11(0)	148(7)		31(2)	
	NaBH_4_ 33–256 °C			NaBH_4_ 33–447 °C	NaBH_4_ 33–212 °C		KBH_4_ *T* = 33–289 °C	
*a*	71(1)			72(1)	76(2)		86(0)	

**Figure 7:**
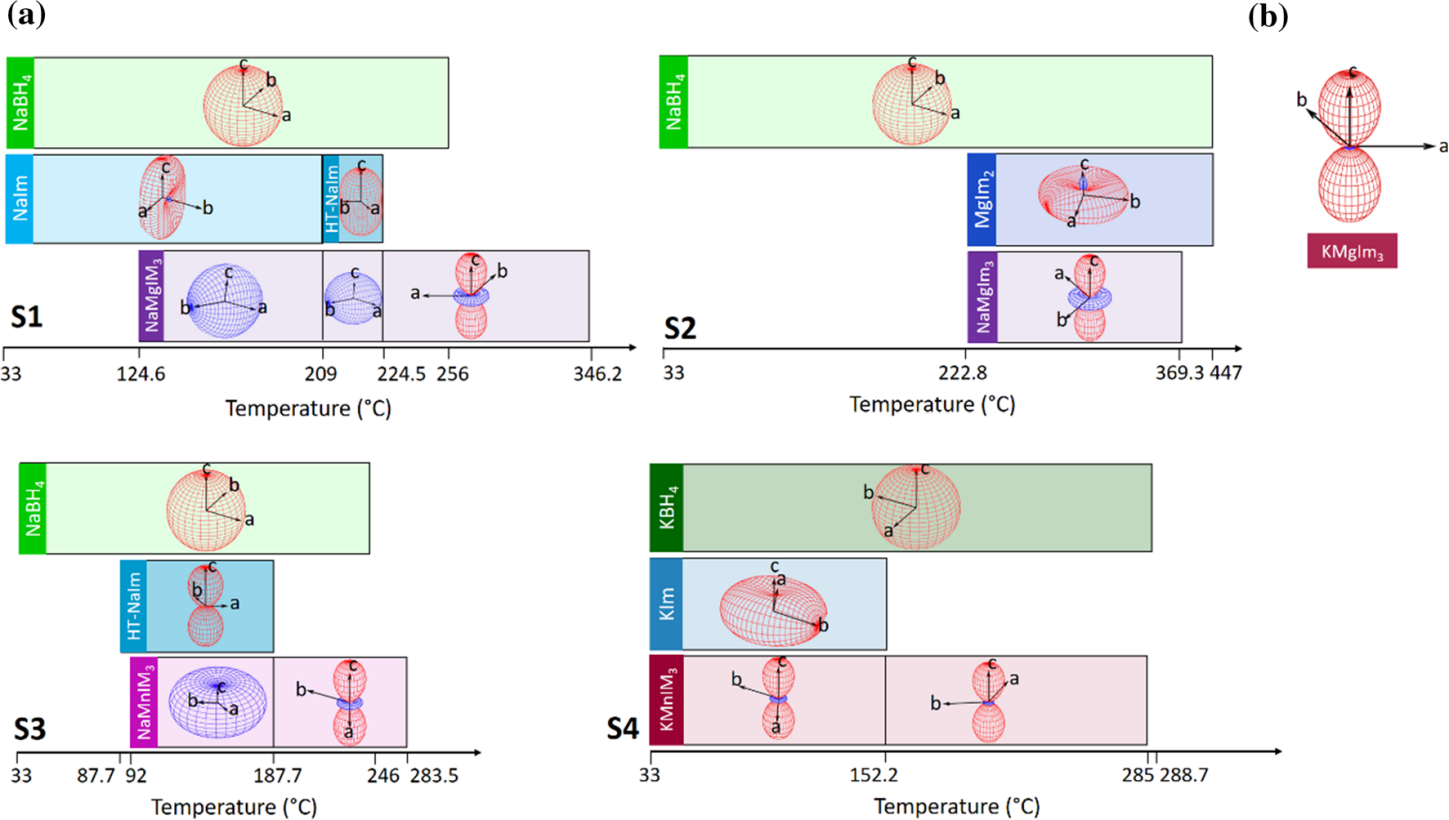
Thermal indicatrices of phases present in samples **a** S1-S4, **b** KMgIm_3_

Thermal indicatrices for each crystalline phase in samples S1-S4 are shown in Fig. 7. Thermal expansion often exhibits similar trends typical of each type of structure. It can be seen that sodium- and potassium- borohydrides (present in samples S1-S3 and S4, respectively) exhibit large positive isotropic linear thermal expansions with large volumetric expansion in the range 217(2) × 10^−6^ K^−1^ < *α*_*V*_ < 265(2) × 10^−6^ K^−1^, which is comparable to the thermal expansion values reported for other metal borohydrides (*α*_*V*_ = 260 − 290 × 10^−6^ K^−1^ for LiBH_4_) [63].

In addition, magnesium and sodium imidazolates (contained in samples S1 and S2, respectively) exhibit similar thermal behaviour. Both exhibit large positive expansion along two directions accompanied by a uniaxial negative thermal expansion in the third direction. A similar shape of thermal indicatrix is found in the case of the potassium imidazolate present in sample S4; large positive expansions along two directions are this time, accompanied by a small positive expansion along the third axis. On the other hand, the high-temperature polymorph of NaIm shows a different behaviour from the room-temperature phase; the HT phase exhibits a small expansion along two directions coupled with a large expansion along the third direction. All compounds show a positive overall volume expansion despite a uniaxial negative expansion in the case of MgIm_2_ and NaIm; of all, KIm exhibits the largest volume expansion α_V_ = 196(9) × 10^−6^ K^−1^.

A particularly interesting thermal behaviour was observed for AMIm_3_ (A = Na, K; M = Mg, Mn), present in samples S1-S4; Fig. 7 shows that in some cases the expansion of bimetallic imidazolates is not uniform over the entire temperature range yet it abruptly changes from colossal negative values of overall volume expansion to moderate positive overall volume expansion. Since the turning point of NaMgIm_3_ (in sample S1) at *T* ~ 224 °C also represents the temperature at which the crystalline HT-NaIm is no longer present in the sample, it is clear that the thermal behaviour of the bimetallic imidazolate is significantly affected by the additional crystalline phases in each sample. Before elaborating the reasons for such a behaviour, it is worth discussing the thermal behaviour of isostructural KMgIm_3_ reported in our previous work [40]. The thermal behaviour of KMgIm_3,_ present in the sample without any other crystalline phases, is shown in Fig. 7b. Indeed, comparing the indicatrix of KMgIm_3_ with the indicatrices of NaMgIm_3_, NaMnIm_3_ and KMnIm_3_ in samples S1-S4_,_ it is clear that all bimetallic imidazolates AMIm_3_ (A = Na, K; M = Mg, Mn) show large positive expansion along the *c*-direction coupled with small biaxial negative expansions, over a certain temperature range (above ~ 220 °C for NaMgIm_3,_ above ~ 180 °C for NaMnIm_3_ while KMnIm_3_ exhibits such a behaviour over the entire temperature range).

**Figure 8:**
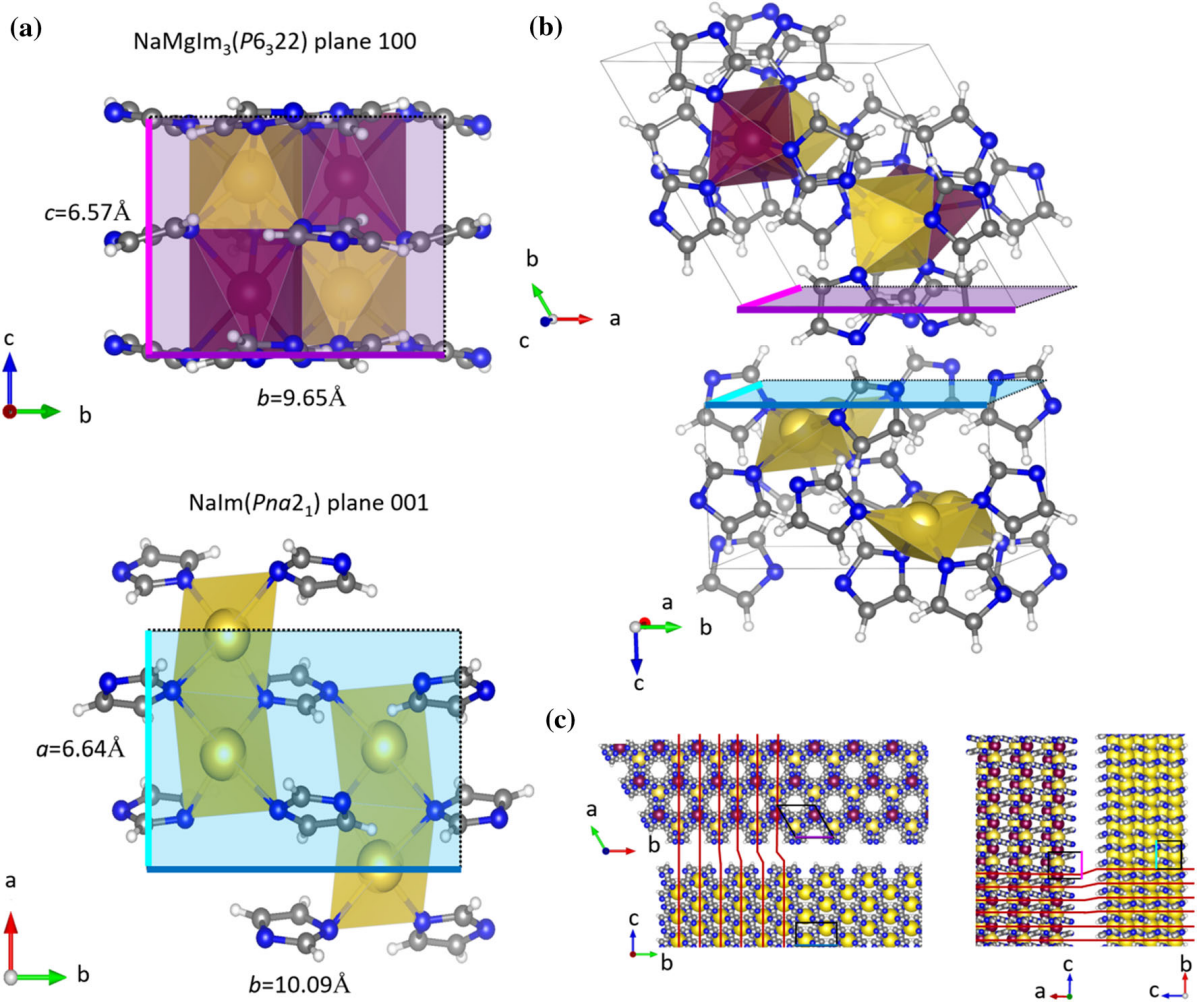
**a** Epitaxial relationship between planes 100 (NaMgIm_3_) and 001 (NaIm), **b** Intergrowth interface of 100 NaMgIm_3_ and 001 NaIm, **c** Lattice mismatch between NaMgIm_3_ and NaIm

Let us now return to the temperature range where NaMIm_3_ (M = Mg, Mn) experiences a large negative expansion in all three directions. Given the phase composition of samples S1 and S3, a plausible explanation for such a behaviour seems to be related to the composite formation and coherent intergrowth of NaMIm_3_ with either NaIm, HT-NaIm or NaBH_4_. However, it is be noted that both NaMgIm_3_ and NaMnIm_3_ abruptly change their expansion trend once crystalline NaIm and/or HT-NaIm are no longer present in the sample suggesting that the intergrowth of bimetallic imidazolate and alkali imidazolate may be responsible for the observed change of thermal expansion behaviour. The intergrowth of NaMgIm_3_ and NaIm in the temperature range *T* = 124.6 − 209 °C was studied in detail; by carefully examining the structures present in sample S1, an epitaxial relationship between the 100 plane of hexagonal NaMgIm_3_ and the 001 plane of orthorhombic NaIm was found and shown in Fig. 8.

**Figure 9:**
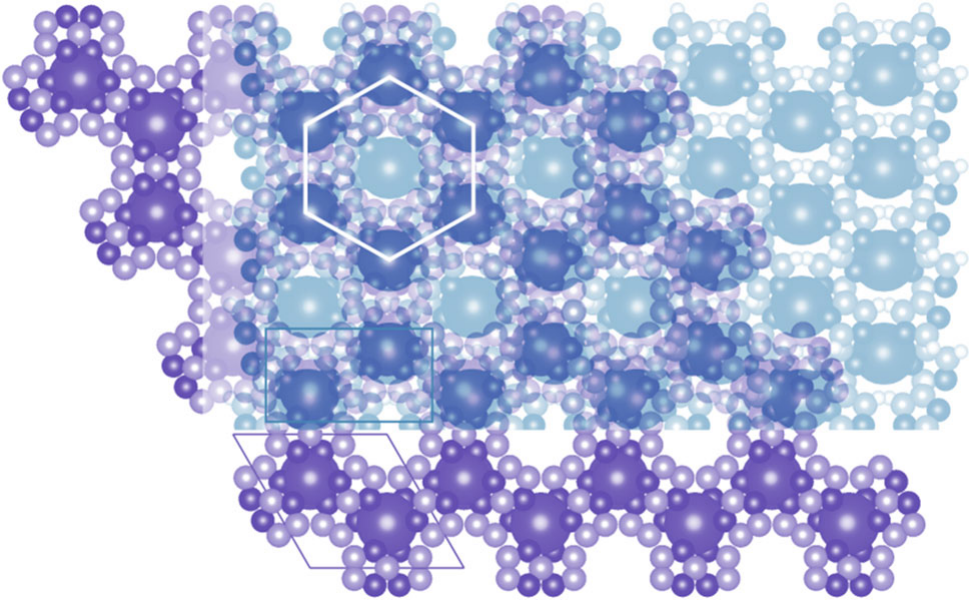
Overlapped coherently intergrown 100 plane of NaMgIm_3_ and 001 plane of NaIm

It can be seen that the *b*-parameters of NaMgIm_3_ and NaIm are similar. Also, the *c* parameter of NaMgIm_3_ is closely related to the *a* parameter of NaIm. Figure 8b shows the 100–001 interface enabling coherent intergrowth of two phases. In general, an interface can be coherent, semi-coherent or incoherent, depending upon the lattice misfit at the interface. If the adjacent layers are assumed to consist of two crystalline phases, 1 and 2, and that both materials are elastically deformable, then there is lattice mismatch due to the difference in lattice constants but the interfaces are still coherent:

$$ f_{1/2} = \frac{{d_{hkl,2} - d_{hkl,1} }}{{d_{hkl,1} }}\quad {\text{and}}\quad f_{2/1} = \frac{{d_{hkl,1} - d_{hkl,2} }}{{d_{hkl,2} }} $$

where *f*_1/2_ is the mismatch of phase 1 relative to phase 2 and *f*_2/1_ the mismatch of 2 relative to 1. The mismatch between coherent phases is often small, typically less than ± 5% [64, 65]. Calculated lattice mismatches at the interface between NaMgIm_3_ and NaIm at different temperatures (130 °C − 180 °C) are small, ranging from 1.6 to ± 4.8%, therefore enabling the formation of coherent interface. At *T* = 162 °C, the lattice parameters of NaMgIm_3_ amount to *b* = 9.613 Å and *c* = 6.654 Å and those of NaIm *b* = 10.037 Å, *a* = 6.769 Å. The lattice mismatch, as shown in Fig. 8c, amounts to + 4.4% along the *b*-direction and + 1.7% along the *c*-direction of NaMgIm_3_ (i.e. *b*-direction of NaIm). Despite the observed lattice mismatch, Fig. 9 shows well-formed coherent intergrowths between the *bc* plane of NaMgIm_3_ (purple) and the *ab* plane of NaIm (turquoise).

From the lattice mismatch, a tensile strain ε_0_ was calculated according to the formula: ${f}_{1/2}\approx -{f}_\frac{2}{1} \approx 2{\varepsilon }_{\mathrm{0,1}}= -2{\varepsilon }_{\mathrm{0,2}}=2{\varepsilon }_{0}$. In the *c*-direction of NaMgIm_3_ (i.e. *b*-direction of NaIm), the tensile strains is + 0.85%, while the strain in the *b*-direction equals 2.2%. The elastic properties in both phases are assumed to be similar, so that the interface strain will be divided approximately in equal parts by both adjacent phases: ε_0,1_ ≈ −ε_0,2_ [64]. Upon further temperature increase, above 209 °C, NaIm transforms to its HT polymorph. Similar to NaIm, NaMgIm_3_ forms an intergrowth relationship with the HT-polymorph of NaIm which continues to affect the thermal expansion behaviour in a similar manner. Above 224.6 °C, once the crystalline HT-NaIm is no longer present in the samples, NaMgIm_3_ begins to exhibit thermal expansion behaviour that is intrinsic to the structure of bimetallic imidazolates. A similar effect of composite coherent intergrowth can be noticed in sample S3. In the temperature range *T* = 92 − 187.7 °C NaMnIm_3_ exhibit negative thermal expansion along all three directions as a consequence of the intergrowth with HT-NaIm. Once the crystalline HT-NaIm is no longer present in the sample, NaMnIm_3_ begins to show a large positive expansion along the *c*-direction that is coupled with small biaxial negative expansions along the *a* and *b*-axis.

The results shown in Fig. 7, also show that NaMgIm_3_ in sample S2 (coexisting with MgIm_2_) exhibits the same trend of thermal expansion (throughout the temperature range from 222.8 to 369.3 °C) as in sample S1 once the crystalline sodium imidazolate is no longer present in the sample (above 224.5 °C). This means that MgIm_2_ and NaMgIm_3_ do not form any epitaxial-like relationship that could affect their thermal expansion. The same was found for KMnIm_3_ in sample S4; KMnIm_3_ exhibit similar expansion behaviour regardless of the presence/absence of the crystalline KIm in the sample. The reason for this is probably too great a difference in the crystal structures of AMIm_3_ (A = Na, K; M = Mg, Mn) compounds and MgIm_2_ or KIm, while sodium imidazolates have comparable structures to AMIm_3_ (A = Na; M = Mg, Mn).

Finally, it is important to address the values of linear thermal expansion. As shown in Table 5**,** the sodium bimetallic imidazolates, when they do not experience any epitaxial relationship, show a small negative expansion along the *a*-axis, ranging from α_*a*_ =  − 9(2) $\times$ 10^–6^ K^−1^ (in the case of NaMgIm_3_) to α_*a*_ =  − 16(1) $\times$ 10^–6^ K^−1^ (for KMnIm_3_), which is accompanied by a moderate to large positive expansion along the *c*-axis, ranging from α_*c*_ = 34(5) $\times$ 10^–6^ K^−1^ (for NaMgIm_3_) to α_*c*_ = 117 $\times$ 10^–6^ K^−1^ (for KMnIm_3_). On the other hand, when bimetallic imidazolates coexist with phases that enable the coherent intergrowth, their thermal expansion coefficients abruptly shift to colossal negative values along all axes for example α_*a*_ =  − 181(8) $\times$ 10^–6^ K^−1^ and α_*c*_ =  − 210(8) $\times$ 10^–6^ K^−1^ in the case of NaMgIm_3_.

### Microstructure

**Figure 10:**
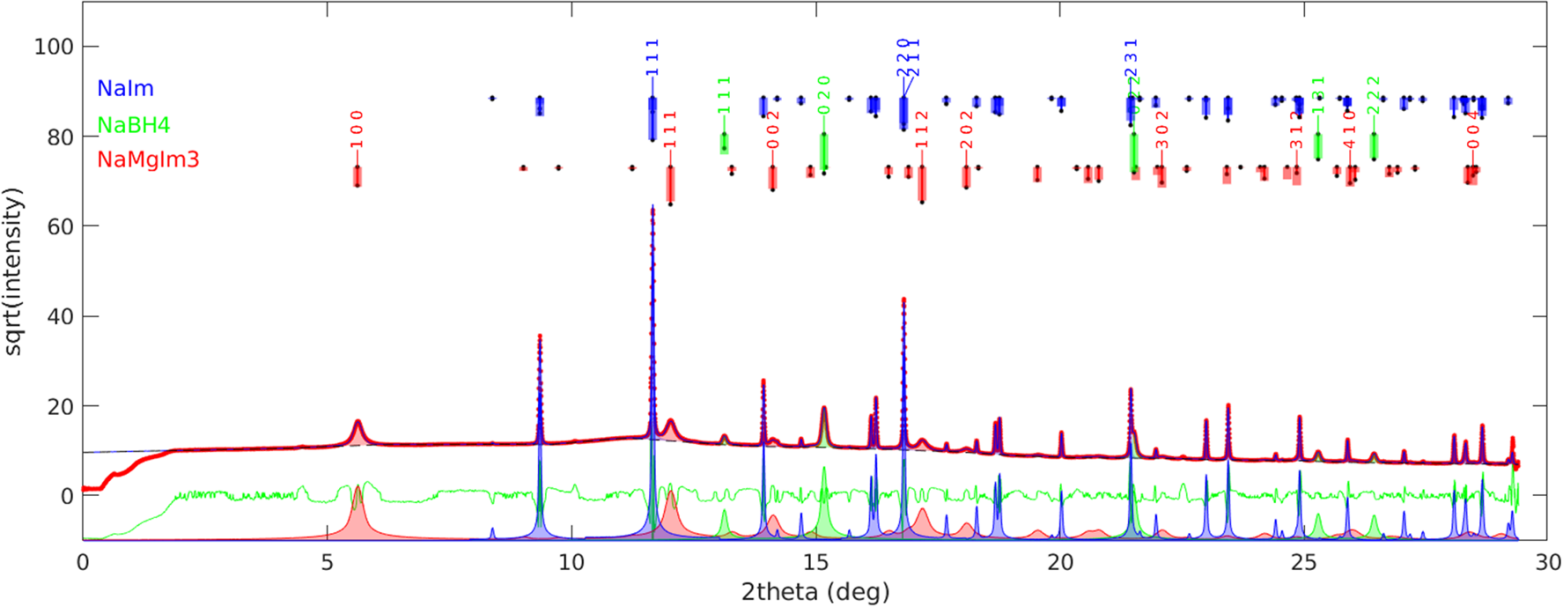
XRD pattern for S1 collected at temperature *T* = 127 °C and fitted by WPPM method in order to obtain microstructure parameters. The individual contributions from different phases on the final pattern are plotted on the baseline of the graph

Heat treatment did not only affect lattice parameters but also microstuctural features of the crystalline phases. To correlate the microstructure parameters with the thermal expansion behaviour of NaMgIm_3_ in S1, we performed a Rietveld/WPPM refinement with program MSTRUCT for collected *in-situ* XRD patterns of S1. The typical fitted pattern is shown in Fig. 10.

In the whole temperature range, the peak width of the NaIm phase corresponds to the instrumental broadening implying negligible microstrain (below 0.1%) and a large crystallite size above the detection limit (300 nm). The same is true for the high temperature polymorph of NaIm. The crystallites of NaBH_4_ also did not exhibit detectable microstrain, however we observed a linear dependence of the crystallite size on temperature, explicitly ranging from 10 nm at room temperature to about 100 nm at 200 °C. Above 200 °C, the crystallite sizes abruptly increased beyond the detection limit.

**Figure 11:**
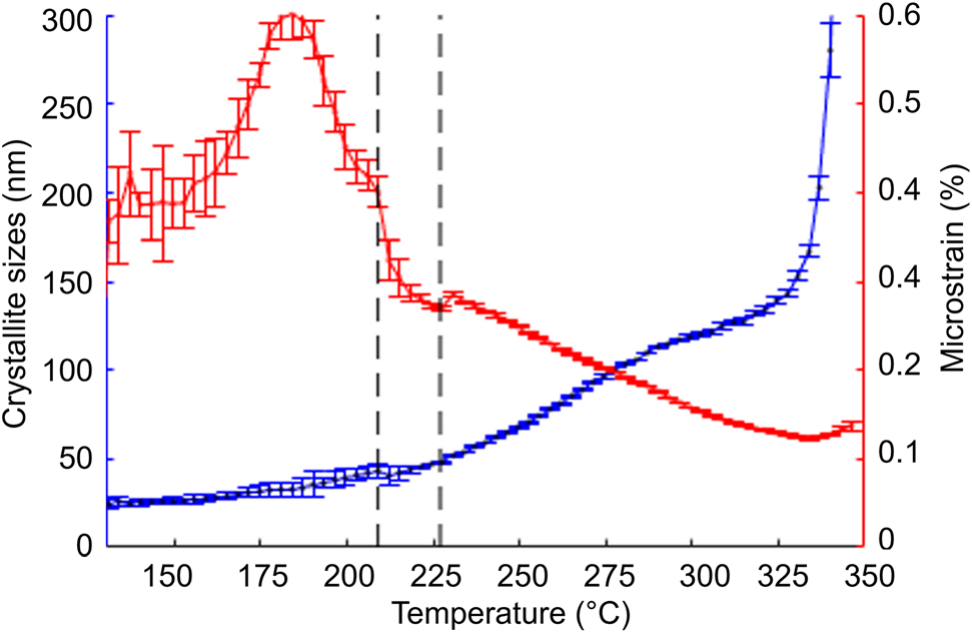
Temperature dependence of the crystallite size (blue) and the microstrain (red) for NaMgIm_3_ phase in S1. Vertical dashed lines located at temperatures 209 °C and 227 °C indicate temperature regions with different thermal expansion behaviour

On the other hand, the microstructure of NaMgIm_3_ evolved quite specifically in all three temperature ranges with different thermal expansion behaviour, as shown in Fig. 11. In the first temperature range (up to 209 °C), where the thermal expansion has the highest negative values, the crystallite sizes grow rather slowly and linearly from  25 to ~ 40 nm. At the same time, the microstrain, which occurs at the very beginning of the phase formation, increases and reaches its maximum at 185 °C. If one recalls Fig. 1, this is the temperature at which NaMgIm_3_ is fully formed and further consumption of NaIm is almost stopped. From this point on, the microstrain is slowly released back to initial value. Further change in trend occurs at the very temperature at which NaIm undergoes the phase transformation to its high-temperature phase. Suddenly, the microstrain in NaMgIm_3_ decreases steeply, while the crystallite size growth rate of remains unaffected.

In the last temperature range, when NaIm melts, both the microstrain and the crystallite size of NaMgIm_3_ followed more or less linear trend. As one would expect for annealing connected with crystal quality improvement, the microstrain was decreasing, while the crystallites were growing. Moreover, the slope of crystallite size growth rate was suddenly higher which could be an indication that the NaIm crystallites slowed down this process before they were melted.

Finally, it should be stressed out that the microstrain is defined as a relative width of the interplanar-distances distribution and this variance can originate from various reasons. However, such possible source in case of very small particle can be an inhomogeneous strain field being a result of interplay between elastically relaxed parts of surface and parts of surface coherently intergrown with mismatched lattice of other extraneous particle. The idea of intergrowth is strongly supported by the fact that the microstrain evolution observed in the NaMgIm_3_ clearly correlates with the following: firstly, the rate of the reaction where NaMgIm_3_ is formed and NaIm is consumed, and secondly with the temperature of phase transformation of NaIm into its high temperature phase. Moreover, the microstrain is significantly released when the NaIm particles are being melted.

## Conclusions

In the search for borohydride-imidazolate frameworks, we have discovered novel bimetallic imidazolates AMnIm_3_ (A = Na, K). Thermal expansion of isostructural AMIm_3_ (A = Na, K; M = Mg, Mn) compounds was studied in detail. An abrupt change in the thermal expansion of NaMIm_3_ (M = Mg, Mn) was noticed when the composite with NaIm and/or HT-NaIm forms; the thermal volume expansion coefficient changes from moderately positive volume expansion for pure bimetallic imidazolate (α_*a*_ = −9(2) $\times$ 10^–6^ K^−1^, α_*c*_ = 34(5) $\times$ 10^–6^ K^−1^) to colossal negative values when composite is formed (α_*a*_ = −181(8) $\times$ 10^–6^ K^−1^ and α_*c*_ = −210(8) $\times$ 10^–6^ K^−1^). Also, it is important to notice that composite material exhibit thermal expansion that is dramatically different from thermal expansion of both of its constituents. This work demonstrates that synthesis of materials containing coherent composites, together with variation of their volume fractions in the composite, can open the way for targeted design of zero thermal expansion materials.

## Supplementary Information

Below is the link to the electronic supplementary material.

Supplementary file1 (DOCX 276 kb)

## Data Availability

CCDC 2126064-2126065 contain the supplementary crystallographic data for this paper. These data can be obtained free of charge via www.ccdc.cam.ac.uk/data_request/cif, or by emailing data_request@ccdc.cam.ac.uk, or by contacting The Cambridge Crystallographic Data Centre, 12 Union Road, Cambridge CB2 1EZ, UK; fax: + 44 1223 336033.
