# Supplementary material for: Abrupt change from moderate positive to colossal negative thermal expansion caused by imidazolate composite formation
Source: J Mater Sci. 2022 Jun 20;57(25):11563–81. doi: 10.1007/s10853-022-07360-z (PMC9246808; doi:10.1007/s10853-022-07360-z)
Supplement: Supplementary file 1 — Supplementary file1 (DOCX 276 kb) [file 10853_2022_7360_MOESM1_ESM.docx]

Journal: Advanced composites and hybrid materials

**Supporting information**

**Abrupt change from moderate positive to colossal negative thermal expansion caused by imidazolate composite formation**

Sanja Burazer, Lukáš Horák, Yaroslav Filinchuk, Radovan Černý*, Jasminka Popović*


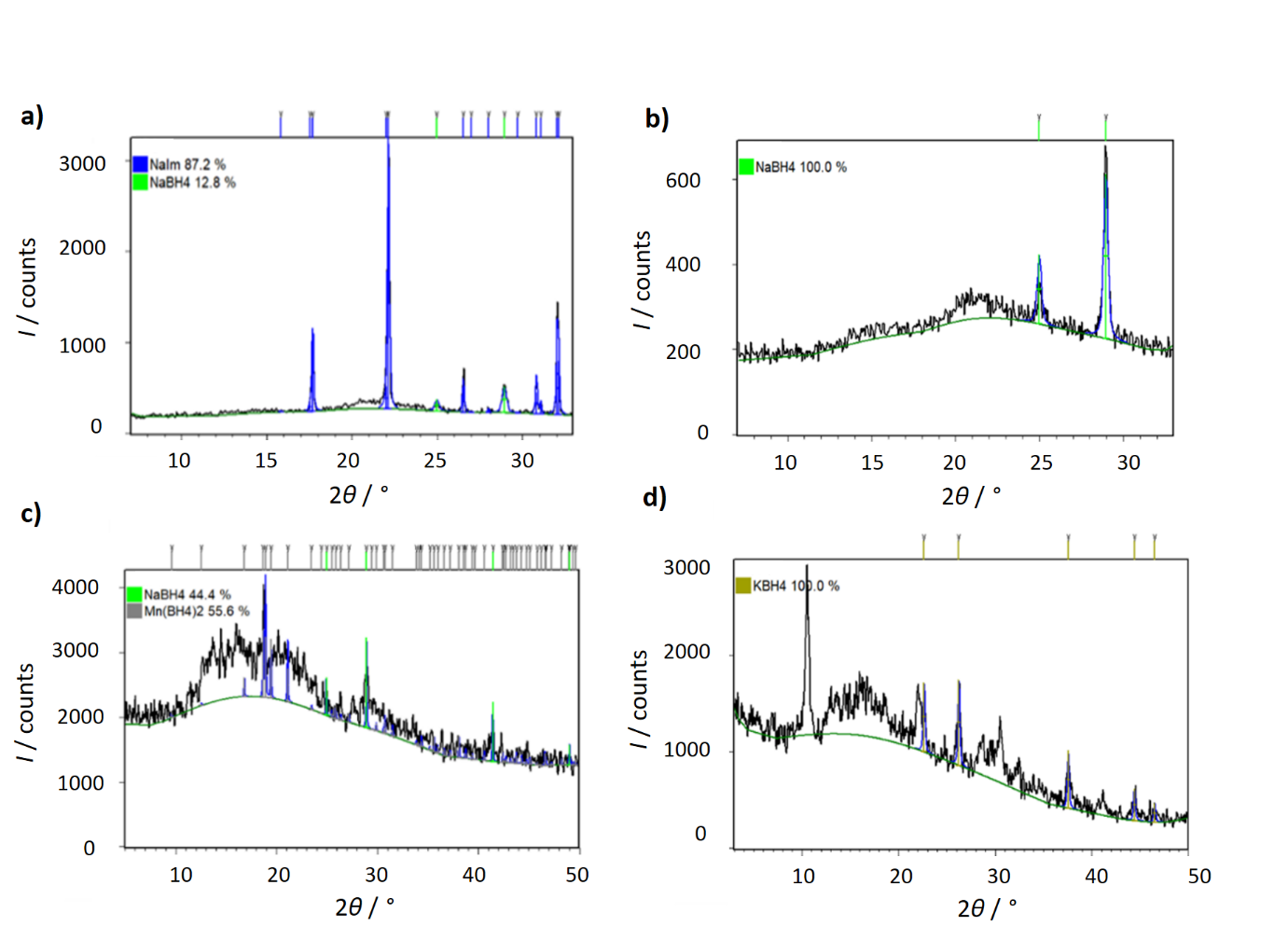


Figure S1. PXRD data collected on laboratory diffractometer at RT for samples a) S1, b) S2, c) S3 and d) S4. Experimental diffraction data are shown in black, while calculated patterns are given in blue. Diffraction lines position of NaBH_4_ are represented by green vertical marks, NaIm by dark blue, Mn(BH4)_2_ by grey, while the vertical marks in the case of KBH_4_ are colored khaki.

The part equations that include all components present in the samples (including those that does not react with other phases):.

**S1**

Mg(BH_4_)_2_ + 6NaIm → 2NaBH_4_ + 4NaIm + MgIm_2 (amorphous)_ [S1]

2NaBH_4_ + 4NaIm + MgIm_2 (amorphous)_ → NaMgIm_3_ + 3NaIm + 2NaBH_4_ [S2]

**S2**

Mg(BH_4_)_2_ + 2NaIm → 2NaBH_4_ + MgIm_2 (amorphous)_ [S3]

4NaBH_4_+3MgIm_2(amorph.)_→ NaMgIm_3_+MgIm_2_+2NaBH_4_+NaIm_(amorph.)_+Mg(BH_4_)_2(amorph.)_ [S4]

**S3**

Mn(BH_4_)_2_+6NaIm→ 2NaBH_4_ + 4NaIm_(amorphous)_  + MnIm_2 (amorphous)_ [S5]

2NaBH_4_ + 4NaIm_(amorphous)_  + MnIm_2 (amorphous)_ → NaMnIm_3_ + 3HT-NaIm + 2NaBH_4_  [S6]

**S4**

Mn(BH_4_)_2_ + 6KIm → 2KBH_4_ + KMnIm_3_ + 3KIm [S7]
